# Targeted Delivery of Neural Stem Cells to the Brain Using MRI-Guided Focused Ultrasound to Disrupt the Blood-Brain Barrier

**DOI:** 10.1371/journal.pone.0027877

**Published:** 2011-11-16

**Authors:** Alison Burgess, Carlos A. Ayala-Grosso, Milan Ganguly, Jessica F. Jordão, Isabelle Aubert, Kullervo Hynynen

**Affiliations:** 1 Imaging Research, Sunnybrook Research Institute, Toronto, Ontario, Canada; 2 Brain Sciences, Sunnybrook Research Institute, Toronto, Ontario, Canada; 3 Laboratorio de Patologia Celular y Molecular, Centro de Medicina Experimental, Instituto Venezolano de Investigaciones Cientificas, Caracas, Venezuela; 4 Laboratory Medicine and Pathobiology, University of Toronto, Toronto, Ontario, Canada; 5 Medical Biophysics, University of Toronto, Toronto, Ontario, Canada; Boston University School of Medicine, United States of America

## Abstract

Stem cell therapy is a promising strategy to treat neurodegenerative diseases, traumatic brain injury, and stroke. For stem cells to progress towards clinical use, the risks associated with invasive intracranial surgery used to deliver the cells to the brain, needs to be reduced. Here, we show that MRI-guided focused ultrasound (MRIgFUS) is a novel method for non-invasive delivery of stem cells from the blood to the brain by opening the blood brain barrier (BBB) in specific brain regions. We used MRI guidance to target the ultrasound beam thereby delivering the iron-labeled, green fluorescent protein (GFP)-expressing neural stem cells specifically to the striatum and the hippocampus of the rat brain. Detection of cellular iron using MRI established that the cells crossed the BBB to enter the brain. After sacrifice, 24 hours later, immunohistochemical analysis confirmed the presence of GFP-positive cells in the targeted brain regions. We determined that the neural stem cells expressed common stem cell markers (nestin and polysialic acid) suggesting they survived after transplantation with MRIgFUS. Furthermore, delivered stem cells expressed doublecortin *in vivo* indicating the stem cells were capable of differentiating into neurons. Together, we demonstrate that transient opening of the BBB with MRIgFUS is sufficient for transplantation of stem cells from the blood to targeted brain structures. These results suggest that MRIgFUS may be an effective alternative to invasive intracranial surgery for stem cell transplantation.

## Introduction

Significant progress in the field of stem cell therapy for neurodegenerative diseases, brain injuries, and ischemic stroke highlights its great potential and remaining challenges [1], [2]. One of the important findings is that neural stem cells transplanted into the brain can survive long term and exert positive effects on the symptoms of disease [3]. For example, in a series of open-label clinical trials where human fetal stem cells were grafted into patients with Parkinson's disease, significant improvements in motor function and timing were observed [3]–[5].

In an animal model of Parkinson's disease, grafted mesenchymal cells have a neuroprotective effect on remaining dopaminergic neurons [6]. Also, grafted neural stem cells integrated into the brain and were found to restore motor function [7]. Recently, neural stem cell transplantation was shown to improve cognition in mouse models of Alzheimer's disease [8]. Furthermore, stem cells have been shown to dramatically improve functional recovery in models of ischemic stroke [1].

One major limitation for the translation of these potential stem cell therapies to clinical practice is the risk associated with invasive cell transplantation methods and the limitation of unwanted repeated surgeries. Intracerebral transplantation of stem cells is the most commonly used method of stem cell delivery to the brain. There are many risks associated with this invasive method, such as risks of surgery, direct tissue trauma causing inflammation and edema [9] as well as graft rejection from immunological response [10]. Other methods to circumvent the risks of surgical transplantation such as intranasal delivery have been proposed but they are untargeted, requiring the cells to migrate to the appropriate brain regions [11]. Intraarterial infusion of hyperosmotic solutions like mannitol, effectively disrupt the BBB and are a potential method for improving stem cell delivery [12]. However, these agents may have serious side effects as they allow potentially cytotoxic compounds present in the blood direct access to the entire CNS for long periods of time.

To circumvent the problems associated with invasive surgeries and to provide localized delivery of stem cells to specific brain regions, we investigated the potential of MRIgFUS to deliver stem cells injected into the bloodstream to the brain. Advances in FUS technology have been used to transiently increase the permeability of the BBB, allowing agents to cross from the blood stream to the brain [13]. FUS applies concentrated acoustic energy on a focal spot measuring a few millimeters in diameter [13]. A microbubble contrast agent is administered systemically and when FUS is applied transcranially to a specific location, the circulating microbubbles begin to oscillate. This leads to changes in the blood vessel wall and a transient increase in the permeability of the BBB [14]. Previous work has shown that transient changes in BBB permeability by FUS allows entry of chemotherapeutics and therapeutic antibodies to targeted areas of the brain [15], [16].

In this study, we demonstrate that FUS-induced BBB disruption allows neural stem cells to move from the blood stream into the brain tissue. Furthermore, using MRI guidance, we were able to target specific, clinically relevant structures for BBB disruption as well as confirm the entry of iron-loaded stem cells. Finally, our results show that the neural stem cells survived well up to 24 hours after FUS indicating that this technique is a powerful option for non-invasive, targeted delivery of neural stem cells to the brain.

## Results

### MRIgFUS increases BBB permeability and permits entry of neural stem cells to the brain

**Figure 1:**
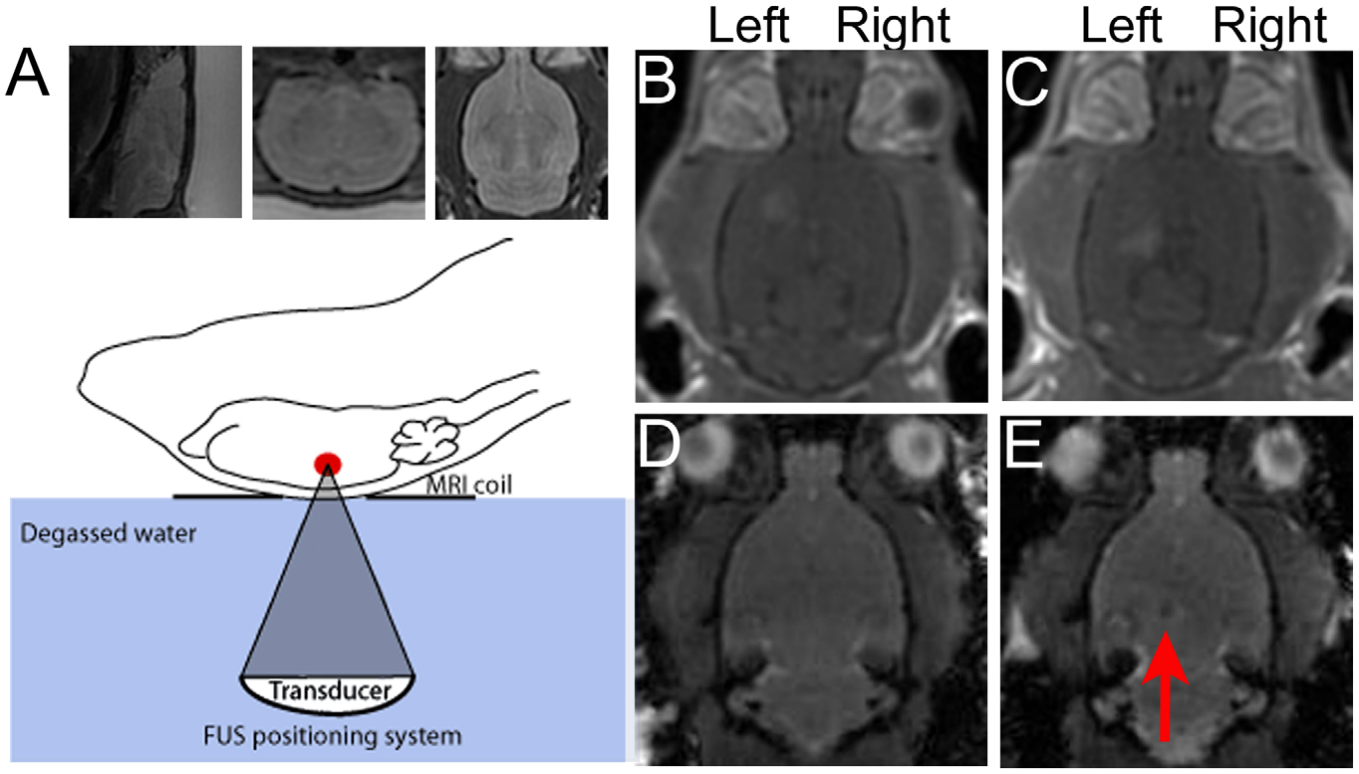
MRIgFUS increases BBB permeability to allow neural stem cells into the brain. A) T2-weighted MR images were taken in all 3 planes (sagittal, coronal and axial) and used to choose precise coordinates in the left striatum and left hippocampus for sonication with FUS. Contrast enhanced T1-weighted MR images were used to confirm increased BBB permeability in the striatum (B) and the hippocampus (C) prior to injection of neural stem cells into the carotid artery. Fast gradient echo (FGRE) sequences taken before (D) and 15 min after sonication (E) were used to confirm the entry of iron-labeled neural stem cells. Dark spots (red arrow) in the MR images taken after cell injection were used to confirm the presence of iron-labeled cells in the tissue.

A schematic of the experimental setup is depicted in Fig. 1A. Briefly, axial, coronal and sagittal T2-weighted MR images were used to precisely target the regions of the striatum and the hippocampus in the left hemisphere of adult rats. Four target points creating a 1 mm square were chosen in each of the striatum and the hippocampus. Microbubbles were injected intravenously immediately prior to the onset of sonication (558 kHz transducer, 0.24 MPa estimated *in situ* pressure, 1 ms bursts, 1 Hz pulse repetition frequency, 120 s total exposure duration) according to Jordão et al [16]. Following the sonication, MR contrast agent was injected through the tail vein to monitor changes in BBB permeability in contrast-enhanced T1-weighted images. Enhancement was observed in the targeted regions of the left striatum (Fig. 1B) and the left hippocampus (Fig. 1C). Analysis of the T1-weighted images revealed that the levels of contrast-enhancement ranged from 18–30% except for one animal where enhancement in the striatum reached 41% (Table S1). GFP-tagged neural stem cells were injected through a catheter in the carotid artery. In order to visualize the translocation of the cells using MRI, we incorporated superparamagnetic iron oxide into the neural stem cells prior to the experiment. The percentage of stem cells containing iron oxide ranged from 80–93%. The addition of the iron oxide did not affect the survival or proliferation rates of the cells in culture (Figure S1). We evaluated whether we could confirm entry of iron-loaded stem cells into the brain using fast gradient echo (FGRE) imaging sequences (TE: 20, TR:30, flip angle:30). We found disturbances in the FGRE images in the region of sonication after injection of iron-loaded cells (Fig. 1D) which were absent prior to sonication (Fig. 1E). These disturbances were specific to the left hemisphere and absent in animals where the injected cells were not iron-labeled or when the animal was not sonicated. Together the data indicate that MRI may be useful for identifying the entry of iron-loaded cells into the brain and to evaluate the success of MRIgFUS delivery. In future studies, the use of iron-oxide particles will assist in tracking the survival and the migration of stem cells transplanted by MRIgFUS over time, as previously established in animal models and human patients [17].

### Stem cells are present in the brain 24 hours after translocation through the BBB

**Figure 2:**
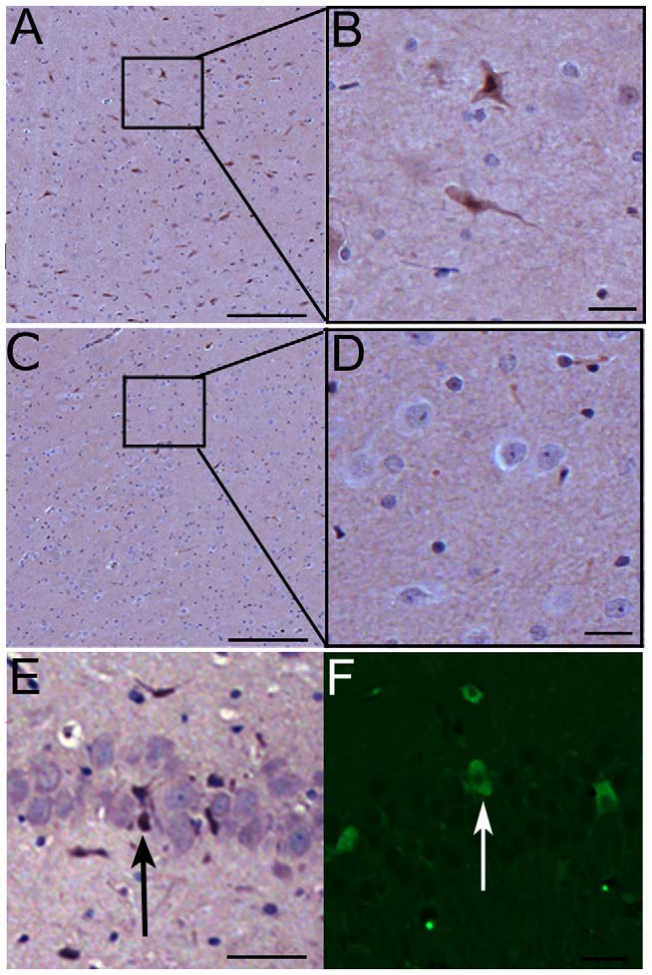
MRIgFUS allows GFP-expressing stem cells to cross the BBB and enter the brain parenchyma. Anti-GFP antibodies were used to detect stem cells in the brain tissue after delivery by MRIgFUS. GFP-positive cells could be detected in the left striatum (A,B) but not in the right, untreated striatum (C,D) indicating the cells were delivered only to the targeted regions. In the hippocampus, stem cells were found in the pyramidal cell layer of the CA1 region (E, arrow) and the granular layer of the dentate gyrus (F, arrow). Scale bars: A,C,E  =  100 µm, B,D,F  =  10 µm.

Animals were recovered and kept for up to 24 hours prior to sacrifice. We then evaluated the success of MRIgFUS for delivery of neural stem cells to the brain using immunohistochemistry on brain sections from animals in each of 3 groups: 1) MRIgFUS + stem cells (n = 8), 2) stem cells alone (n = 4) and 3) MRIgFUS alone (n = 2). GFP-positive stem cells were identified in the left hemisphere of all animals administered stem cells following MRIgFUS (Fig. 2A,B) and were not observed in the right hemisphere, which was untreated with MRIgFUS, (Fig. 2C,D) or in animals given stem cells but no sonication. GFP-positive cells were found in the left hippocampus as well and exhibited a neuronal-like phenotype. The cells were mainly located in the granular layer of the dentate gryus and the pyramidal layer of the CA1 region (Fig. 2E,F). We estimated the total number of stem cells delivered to the brain by counting the number of GFP positive cells in a 1 mm×1 mm region of interest at the hippocampus. We analysed the section from each animal which was determined to be the centre of the sonicated region. Our analysis revealed that 32±8.7 cells were successfully delivered per mm^2^ region of the brain (n = 8).

### Stem cells express neuronal markers in vivo

**Figure 3:**
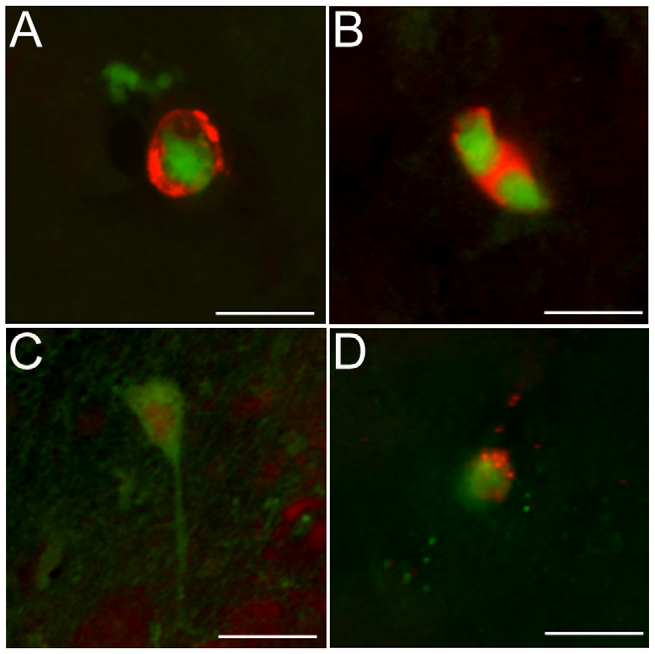
GFP-positive cells express markers of stem cells and immature neurons *in vivo*. Immunohistochemistry revealed GFP-positive cells in the brain expressed polysialic acid (A, red) and nestin (B, red) after 4 hours. After 24 hours, nestin positive cells exhibit a neuronal phenotype (C) and a few GFP-positive cells expressed doublecortin, a marker of immature neurons (D, red). Scale bars: 10 µm.

We demonstrated that GFP-positive cells in the brain also expressed the stem cell markers polysialic acid (Fig. 3A) and nestin (Fig. 3B,C) after 4 hours in *vivo*. The staining pattern was similar to that observed *in vitro* (Figure S1). The MRIgFUS treatment alone did not induce obvious nestin or polysialic acid expression in non-GFP expressing cells at 4 or 24 hours. It has been reported that neural stem cells *in vivo* express neuronal or glial markers within 1–3 days [18], thus, we evaluated the expression of doublecortin, a marker of immature neurons, and glial fibrillary acidic protein (GFAP), a marker of astrocytes. At 24 hours post-sonication, we found GFP-positive cells which exhibited a neuronal phenotype (Fig. 3C) and some which also expressed the neuronal marker, doublecortin (Fig. 3D). We did not find any evidence of GFP-positive cells expressing GFAP 24 hours after sonication. Many stem cells express astrocytic markers when they are capable of differentiating into both mature neurons and astrocytes however the neural stem cells used in these experiments did not express GFAP *in vitro* indicating that the cells are programmed for neuronal lineage. These results suggest that the stem cells survive after transplantation with MRIgFUS and that they are capable of differentiating into a neuronal lineage *in vivo*.

### Histological evaluation showed minimal tissue damage following MRIgFUS

**Figure 4:**
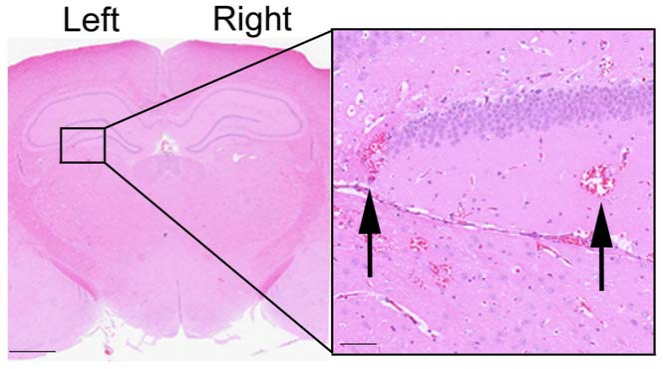
Histological analysis reveals limited tissue damage. Brain sections were processed for H&E according to standard procedures. Red blood cell extravasation was observed in the sonicated regions (inset). There was no indication of lesioning or permanent tissue damage. Scale bars  =  2000 µm, inset  =  100 µm.

Post-mortem histology identified the presence of extravasated red blood cells in the tissue at the site of sonication (Fig. 4). We did not observe any evidence of lesioning or other severe damage to the tissue as has been previously reported [19].

## Discussion

Stem cells have shown great potential for treatment of a variety of brain disorders including neurodegenerative diseases and ischemic stroke [1]–[3]. Currently, the most common method for stem cell delivery to the brain is using intracerebral transplantation which has many associated risks. Here, we present data showing that FUS can alter the BBB to allow neural stem cells to move from the blood stream into the brain parenchyma, providing an alternative to intracerebral transplantation.

Using MRI guidance, we demonstrate that the FUS energy can be concentrated to a region of interest thereby allowing the stem cells to be delivered to a single brain structure, sparing the surrounding brain tissue. In this study we delivered cells to the left striatum and the left hippocampus which are regions of clinical interest for treatment of Huntington's and Alzheimer's disease, respectively. MRIgFUS is advantageous to other methods off BBB opening, such as hyperosmotic agents or vasodilators, which cause widespread BBB permeabilization and have had limited success in translocation of stem cells [12], [19]. The accuracy of MRIgFUS is similar to stereotaxic injection and causes limited tissue damage. Our histological analysis revealed some red blood cells present in tissue around the areas of sonication. Our previous studies in rabbit brain have demonstrated that BBB opening and red blood cell extravasation do not produce long term damage [20]. Since the average diameter of the neural stem cells is approximately 10 µm, we predict that the extent of BBB opening required to allow entry of the stem cells will result in some red blood cell extravasation. We suspect that most of the delivered stem cells enter the brain via widened tight junctions created by FUS, similar to the red blood cells. However, previous reports have suggested that stem cells can also cross the BBB via induced paracellular routes [21]. It will be imperative in future studies to optimize FUS parameters to strike a balance between maximizing the entry of stem cells to the brain and minimizing red blood cell extravasation caused by MRIgFUS.

Previous work has used MRI to monitor the survival and migration of iron-labeled cells in the brain [17], [22]. Here, we investigated whether we could FGRE MR imaging sequences which are sensitive to iron and blood products, to confirm cell delivery after FUS. Indeed, we observed hypointense regions on the images in the sonicated regions after iron-loaded stem cells were delivered with FUS. We did not see corresponding hypointense regions on images from animals who received non-iron loaded stem cells suggesting that the hypointense regions are due to the presence of iron-loaded stem cells in the brain. However, as mentioned, FGRE imaging sequences are also sensitive to the presence of blood and our histological analysis shows red blood cell extravasation occurs at the FUS site. Therefore, we cannot irrefutably conclude that the hypointense region of the FGRE images is the stem cells. Future work to minimize the extent of red blood cell extravasation during stem cell delivery would improve the use of MRI to track cell entry.

One of the concerns arising from intracerebral transplantation of neural stem cells is the immunological reaction and inflammation which can lead to graft rejection [9]. The immunological reaction caused by transplantation of cells with MRIgFUS has not been fully evaluated. In our previous work, we observed edema (detected on T2-weighted MRI images after sonication) when enhancement on T1-weighted images was greater than 30% [23]. In the current study, we demonstrated that stem cells entered the brain in animals showing enhancement of <30% suggesting that edema can be minimized by controlling the sonication parameters and limiting the T1-weighted enhancement. However, the extent of inflammatory and immunological reaction due to MRIgFUS still requires investigation. The increase in BBB permeability has been shown to be transient and reversible [14], so it is reasonable to expect that repeated treatments for multiple transplantations is possible. The use of MRIgFUS to deliver small volumes of stem cells over several weeks or months may decrease the insult of transplantation to the brain, reduce the inflammatory response and improve cell survival *in vivo*.

We demonstrate that MRIgFUS is a powerful technique that may be useful for delivery of stem cells for treatment of ischemic stroke. Specifically, intraarterial delivery of stem cells has shown great potential as a stroke treatment but there is great variability in the number of cells which actually arrive at the site of damage [24]. By combining intraarterial delivery of stem cells with MRIgFUS, the permeability of the BBB in the infarcted region can be increased thereby improving the number of cells with access to the ischemic penumbra. In this study, we chose the carotid artery as the preferred entry point in our rat model however catheterization of the femoral artery is a routine clinical practice and could be used for translation of MRIgFUS to the clinic.

Overall, the delivery of stem cells to the brain with FUS shows promise and it represents several advantages compared to traditional stereotaxic, intracerebral transplantation of cells into the brain. First, FUS does not require invasive intracranial surgery, limiting the associated risks of infection and hemorrhage. Second, the increase in BBB permeability with FUS is transient and non-invasive, indicating it is plausible that patients could receive multiple treatments over their lifetime to improve the outcome of the therapy. Third, for targeting multiple sites, FUS can be used to deliver cells to the striatum and the hippocampus, or other regions, in the same session and with less damage relative to needle implantation. Cell delivery with FUS is targeted and can be used to avoid the distribution of cells along the needle track as often occurs with intracerebral transplantation [1]. Finally, MRI provides excellent soft tissue contrast and therefore can be used to precisely target brain regions of interest, minimizing the distance that stem cells have to migrate *in vivo*. Inadequate cell migration has been a key problem in human patients [25] and thus may limit the clinical relevance of methods where the cells must migrate large distances, such as intranasal cell delivery [11].

The results shown here suggest that MRIgFUS is an effective tool for delivery of neural stem cells to targeted regions of the brain. MRIgFUS is non-invasive, results in minimal tissue damage and has potential for repeated cell delivery. MRIgFUS may be the key to improving cell delivery to the brain and leading to improvements in stem cell therapy in the clinical setting.

## Materials and Methods

### Animals

Sprague Dawley rats (200–250 g) with a vascular catheter in the left common carotid artery were received from Charles River Laboratories (Wilmington MA). All experimental procedures were approved by the Animal Care Committee of Sunnybrook Research Institute (Protocol 290) and conformed to the guidelines set by the Canadian Council on Animal Care and the Animals for Research Act of Ontario.

### Cell culture

GFP-transfected embryonic cortical neural progenitor cells were obtained from Millipore and grown as adherent monolayers on polyornithine (10 µg/mL] and laminin (5 µg/mL) coated Petri dishes. The cells were grown in neural stem cell defined media (supplemented with basic fibroblast growth factor (20 ng/mL), epidermal growth factor (20 ng/mL) and heparin (2 µg/mL)). Superparamagnetic iron oxide (5 µg; Sigma Aldrich) was added to the stem cell media 24 hours prior to the beginning of the experiment. The effect of the uptake of iron cells on cell survival and proliferation was monitored in concurrent *in vitro* experiments. Cultures containing iron oxide were grown for up to 48 hours. At 0, 24 and 48 hours, cells were collected with Accutase, centrifuged and counted with a hemocytometer. Trypan blue was used to exclude dead cells. Total live cell number was graphed and analysed using Graph Pad Prism 5.0. Prior to each *in vivo* experiment, the percentage of cells containing iron oxide was determined with a hemocytometre using light microscopy. For injection *in vivo*, iron-loaded and non iron-loaded cells were treated with Accutase, collected, centrifuged, and re-suspended at a concentration of 2×10^6^ cells/mL in Hanks balanced salt solution (HBSS). Cells were kept at 4°C until they were injected, a maximum of 2 hours later.

### MRIgFUS

Animals were anesthetised with ketamine (50 mg/kg) and xylazine (10 mg/kg) and depilatory cream was used to remove hair from the head. Animals were placed supine on a custom-built FUS positioning system with their heads coupled to a degassed water bag and the entire system was placed in the 1.5T MR scanner (GE Healthcare). Baseline T1-weighted (TE =  10 ms, TR = 500 ms) and fast gradient echo (FGRE) sequences (TE = 20 ms, TR = 30 ms, NEX = 2, flip angle = 30°, slice thickness = 1 mm, matrix = 256×256) in addition to baseline T2-weighted (TE = 60 ms, TR = 2000 ms) images in all 3 planes (coronal, axial and sagittal) were acquired. The T2 images were used to choose 4 precise target points, creating a 1 mm square in each of the striatum and hippocampus. Definity® microbubble contrast agent (0.2 mg/kg, Lantheus Medical) was injected through a tail vein catheter immediately prior to sonications. Sonications were completed using a 558 kHz transducer (0.24 MPa estimated *in situ* pressure, 10 ms bursts, 1 Hz pulse repetition frequency, 120 s total exposure duration) according to Jordão et al [16]. Opening of the BBB was confirmed on a T1-weighted image after injection of an MRI contrast agent (Omniscan, 0.2 mL/kg) through the tail vein. Following BBB opening, the plug from the carotid artery catheter was removed and flushed with 0.5 mL of heparinised saline and then allowed to bleed back from the catheter. Neural stem cells, suspended in 1 mL of HBSS as described above, were injected into the carotid artery catheter followed by 1 mL of saline flush. Animals were sacrificed either 4 or 24 hours after sonication. Animals which were given iron-loaded stem cells (n = 6), were re-imaged 15 and 60 min after sonication. FGRE sequences were used to identify disturbances in the MR images indicative of entry of iron-loaded cells.

### Immunohistochemistry

Rats were deeply anesthetised with ketamine/xylazine and perfused intracardially with saline, followed by 10% formalin. Brains were removed, post-fixed overnight and equilibrated in 30% sucrose. Coronal sections were cut at 50 µm from the beginning of the striatum through the hippocampus. Serial sections were collected in 24 well plates filled with cryoprotectant. Sections were processed using standard immunohistochemistry procedures [26]. Mouse monoclonal anti-nestin (1∶200; Millipore), rabbit anti-GFP (1∶400; AbCAM), goat anti-doublecortin (1∶400; Santa Cruz Biotechnology), rabbit anti-GFAP (1∶4000; Dako), mouse monoclonal anti-PSA (1∶1000, generously provided by Dr. Tatsunori Seki) were applied overnight at 4°C in phosphate buffered saline containing 5% donkey serum and 0.5% Triton X-100. Sections were then incubated with appropriate secondary antibodies (Jackson ImmunoResearch) for 2 hours at room temperature and coverslipped with Vectashield hard-set mounting media (Vector Laboratories). Cells plated *in vitro* were cultured for 24 hours and fixed with methanol for 10 min and then processed as described above.

To identify red blood cell extravasation, brains were routinely processed for paraffin-wax embedding, cut into 4 µm sections and stained with Hematoxylin & Eosin (H&E). To confirm the immunohistochemistry results, sequential 4 µm sections were stained with anti-nestin and anti-GFP antibodies using Vector ImmPRESS staining kits and visualized with 3,3′-diaminobenzidine.

### Analysis

Analysis of MR images was performed in MATLAB (The MathWorks, Natick, MA, USA). Enhancement of the sonication locations relative to baseline was averaged over a 2×2 mm region of interest. The region of interest was selected based on the maximum enhancement observed over a series of contrast-enhanced T1-weighted images. Fluorescent images were collected from thin optical sections throughout the thickness of the tissue using a Zeiss Axiovert confocal laser scanning microscope 510 with a 20× and 63× objectives. Figure montages were created in Adobe Photoshop 7.0 (Adobe Systems Inc, San Jose CA, USA). Stem cells were quantified by counting the number of GFP positively stained cells in a 1 mm×1 mm region of interest in coronal sections. We analyzed the section determined to be at the centre of the sonication location (n = 8). The same region was examined in the contralateral hemisphere in the same coronal section.

## Supporting Information

Figure S1 **Iron-oxide labeling does not affect cell survival, proliferation or differentiation.** A) One million cells were plated on coated chamber slides in the presence or absence of 5 µg superparamagnetic iron oxide (n = 3). Cells were counted at 24 and 48 hours using Trypan blue to exclude dead cells. Total cell counts and rate of proliferation were unchanged by the incorporation of iron into the cells. Data was analyzed using Graph Pad Prism. B) After 24 hours in culture, cells were fixed with methanol for 10 min at room temperature. Standard immunohistochemistry was performed and images were taken with confocal microscopy. GFP-positive cells (left panel) were stained for mouse anti-nestin-Cy3 (middle panel) and the overlay (right panel) shows colocalization demonstrating the stem cells express nestin *in vitro*. Similar results were obtained using anti-polysialic acid.
(TIF)

Table S1 **T1 weighted enhancement levels confirm BBB opening by FUS**. Contrast enhanced T1 weighted MR images were analyzed using MATLAB. Enhancement of the sonication locations was averaged over a 2×2 mm region of interest in the image showing maximum enhancement. The level of enhancement was compared to the same region in the opposite (non-sonicated) hemisphere.
(PDF)
