# Supplementary figures and images for: Targeted Delivery of Neural Stem Cells to the Brain Using MRI-Guided Focused Ultrasound to Disrupt the Blood-Brain Barrier
Source: PLoS One. 2011 Nov 16;6(11):e27877. doi: 10.1371/journal.pone.0027877 (PMC3218061; doi:10.1371/journal.pone.0027877)

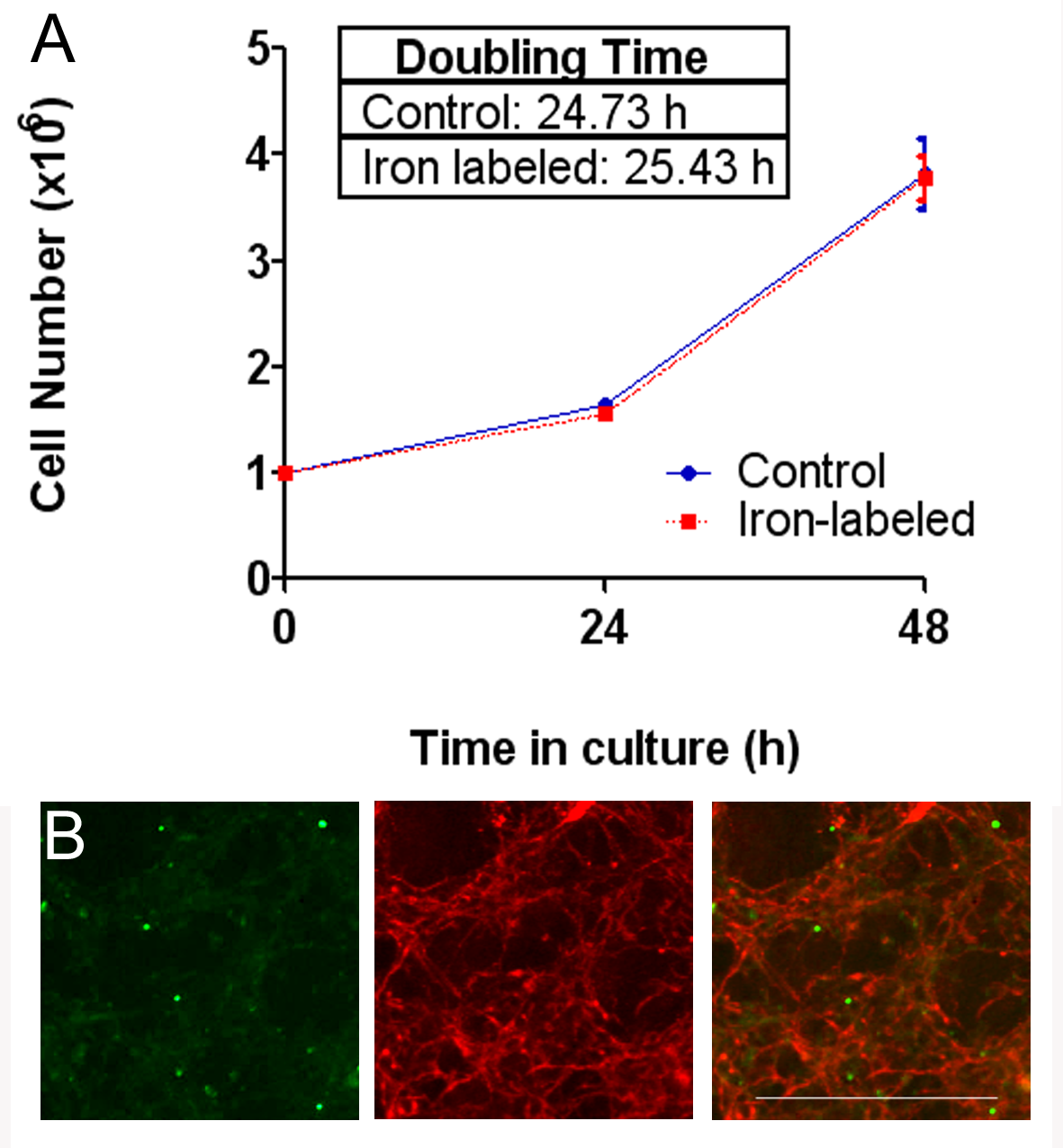

Supplement: Figure S1 — Iron-oxide labeling does not affect cell survival, proliferation or differentiation. A) One million cells were plated on coated chamber slides in the presence or absence of 5 µg superparamagnetic iron oxide (n = 3). Cells were counted at 24 and 48 hours using Trypan blue to exclude dead cells. Total cell counts and rate of proliferation were unchanged by the incorporation of iron into the cells. Data was analyzed using Graph Pad Prism. B) After 24 hours in culture, cells were fixed with methanol for 10 min at room temperature. Standard immunohistochemistry was performed and images were taken with confocal microscopy. GFP-positive cells (left panel) were stained for mouse anti-nestin-Cy3 (middle panel) and the overlay (right panel) shows colocalization demonstrating the stem cells express nestin in vitro. Similar results were obtained using anti-polysialic acid. (TIF) [file pone.0027877.s001.tif]
