# Supplementary material for: Targeted Delivery of Neural Stem Cells to the Brain Using MRI-Guided Focused Ultrasound to Disrupt the Blood-Brain Barrier
Source: PLoS One. 2011 Nov 16;6(11):e27877. doi: 10.1371/journal.pone.0027877 (PMC3218061; doi:10.1371/journal.pone.0027877)
Supplement: Table S1 — T1 weighted enhancement levels confirm BBB opening by FUS. Contrast enhanced T1 weighted MR images were analyzed using MATLAB. Enhancement of the sonication locations was averaged over a 2×2 mm region of interest in the image showing maximum enhancement. The level of enhancement was compared to the same region in the opposite (non-sonicated) hemisphere. (PDF) [file pone.0027877.s002.pdf]

| Animal | Striatum | Hippocampus |
|---|---|---|
| 1 | N/A | 128% |
| 2 | N/A | 119% |
| 3 | N/A | 125% |
| 4 | N/A | 120% |
| 5 | 127% | 118% |
| 6 | 119% | 122% |
| 7 | 118% | 119% |
| 8 | 141% | 128% |
